# Elucidating the Role of Copper-Induced Mixed Phases on the Electrochemical Performance of Mn-Based Thin-Film Electrodes

**DOI:** 10.1021/acsomega.3c05614

**Published:** 2023-11-30

**Authors:** Pramitha Adoor, Shreeganesh Subraya Hegde, Badekai Ramachandra Bhat, Sudhakar Narahari Yethadka, Raviprakash Yeenduguli

**Affiliations:** † Semiconductor and Photovoltaics Lab, Department of Physics, Manipal Academy of Higher Education, Manipal Institute of Technology, Manipal 576104, Karnataka, India; ‡ Catalysis and Materials Chemistry Laboratory, Department of Chemistry, National Institute of Technology Karnataka, Surathkal, Mangalore 575025, Karnataka, India; § Department of Chemistry, Manipal Institute of Technology, Manipal Academy of Higher Education, Manipal 576104, Karnataka, India

## Abstract

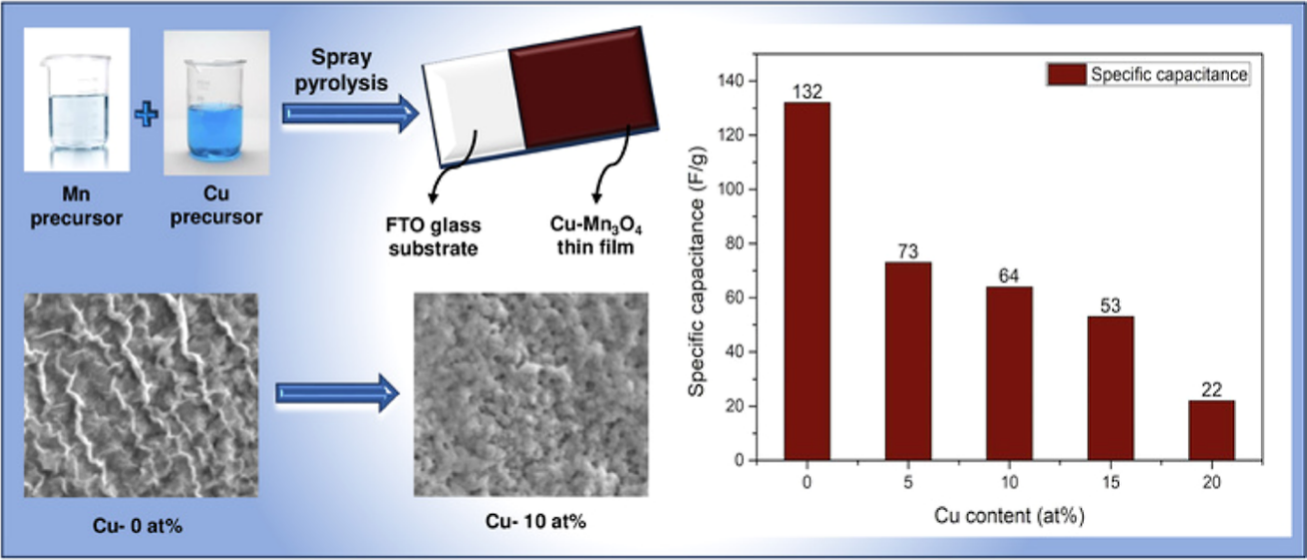

Manganese oxide is a fascinating material for use as a thin-film electrode in supercapacitors. Herein, the consequences of copper incorporation on spray pyrolyzed manganese oxide thin films and their electrochemical performance were investigated. The Cu-incorporated manganese oxide thin films were deposited by spray pyrolysis, and their structural and electrochemical properties were thoroughly evaluated. The formation of the spinel Mn_3_O_4_ phase with effective Cu incorporation was confirmed by X-ray diffraction investigation. Through Raman studies, it was noticed that mixed phases of manganese oxide tend to form after Cu incorporation, and this result was also reflected in X-ray photoelectron spectroscopic studies. The surface morphology and roughness were also altered by the addition of copper. However, electrochemical measurements implied a reduction in the specific capacitance upon copper inclusion. The cyclic voltammetry test indicated a specific capacitance of 132 F/g for Mn_3_O_4_ electrodes, but a substantial drop for copper-incorporated samples due to the mixed manganese phase. The decremental tendency was further supported by galvanostatic charge–discharge studies and electrochemical impedance spectroscopic measurements. These results provide valuable insights into the effects of copper addition in manganese oxide thin-film-based electrodes for energy storage applications.

## Introduction

The upsurge in energy consumption and the resulting energy scarcity have led researchers to the development of energy storage systems. Supercapacitors have recently been identified as viable alternatives for storage systems in high-power applications, which have caught the interest of researchers.^1,2^ To date, materials such as carbon-derived materials, transition metal oxides, hydroxides, sulfides, nitrides, and conducting polymers were explored as an electrode in supercapacitors owing to their superior electrochemical properties.^3,4^ Transition metal oxides (TMOs) are one of the renowned classes of materials that frequently offer significant storage capacity with excellent stability. Their higher energy density compared to carbon-derived materials is attributed to the involvement of faradaic redox reactions.^5^ Among TMOs, manganese oxide is considered as a promising material because of its earth abundance and cost-effectiveness. Moreover, it has an amenability to utilize in diverse applications viz., sensing,^6^ catalyst,^7^ batteries,^8^ etc. Besides, various oxidation states of manganese resulted in different phases, i.e., MnO(II), MnO_2_(IV), Mn_2_O_3_(III), Mn_3_O_4_(II, III), etc. In the aforementioned phases, Mn_3_O_4_ exhibits stability at a high temperature and its mixed valency (Mn^2+^ and Mn^3+^) were available for redox reactions in electrochemical applications.^9^ Even though this material exhibits an optimal property to act as an electrode, the theoretical value of the specific capacitance has not been attained so far. Hence, further in-depth studies are required to enhance the working performance of Mn_3_O_4_-based supercapacitors.^10^

Apart from their bulk form, Mn_3_O_4_ thin films show promising properties that are useful in many applications. In addition, the lower thickness of thin films offers a high surface-to-volume ratio.^11^ Different thin film deposition techniques have been utilized to deposit the Mn_3_O_4_ thin films including sputtering,^12^ electrodeposition,^13^ successive ionic layer adsorption and reaction (SILAR),^14^ spray pyrolysis,^15−18^ etc. Among these, spray pyrolysis is one of the versatile techniques where the thin films are synthesized by the pyrolytic decomposition of the metal salt precursor on the hot substrate.^19^

The electrochemical performance of Mn_3_O_4_ thin films relies on various factors such as oxidation states, secondary phases, surface morphology, defects, etc. Moreover, the synthesis conditions have a vigorous role in determining the properties of Mn_3_O_4_ thin films.^20^ With the aim of improving the performance of Mn_3_O_4_ electrodes, researchers have been utilized various approaches such as nano structuring, doping, forming composites, etc.^21−24^ Incorporating a different element to form mixed oxides is one such strategy that may alter the electrochemical properties depending on the changes the dopant brings in the properties of the material. Thus, far, various elements such as Ni,^25^ Fe,^26^ Cr,^27^ Co,^28^ Zn,^29^ Cu,^7^ etc. were used as dopants for Mn_3_O_4_ to tune their electrochemical properties. Li and others introduced Cu^2+^ into Mn_3_O_4_ through a facile synthesis route. They observed that Cu^2+^ partly substituted the Mn^3+^ in the lattice which brought significant improvements in both electronic conductivity and efficiency of electrochemical storage.^30^ Barai and others synthesized a composite with Cu-doped Mn_3_O_4_:Mn-doped CuO nanostructures through the solution process. They reported that the enhancement in the specific capacitance value of the composite is due to the reduced particle size, higher surface area, increased conductivity, and higher oxygen vacancies.^31^ Chen and others synthesized budlike Cu-doped Mn_3_O_4_ nanostructures using the hydrothermal method. The addition of 1.5 at % of Cu resulted in a porous hollow nanostructure with high surface area which contributed to higher electrochemical performance.^32^ Thus, from the previous studies, it was noticed that Cu can potentially modify the electrochemical properties of the host material owing to their good electronic conductivity and appreciable redox activity. However, most of the studies were reported for Mn_3_O_4_ nanostructures and focused studies are seldom less on Mn_3_O_4_ electrodes as thin films. The binder-free thin film electrodes might show a very good electrochemical performance owing to their very short diffusion path for the electrolyte ions.^33^ However, during the synthesis of the thin film, its growth is restricted to two dimensions; hence, it will exhibit a different property in comparison with its nanostructures.

Even though the Cu-added Mn_3_O_4_ nanostructures show a significant enhancement in electrochemical performance in comparison with the pristine sample, studies on the impact of Cu incorporation on Mn_3_O_4_ films have not been reported so far. Consequently, this study is the first to examine the effects of Cu inclusion on spray-pyrolyzed Mn_3_O_4_ thin films. Furthermore, a wide variety of characterization techniques were employed to determine its various properties. The prominent changes in the structural, morphological, topographical, and electrochemical properties were noticed, and the attained results were discussed extensively, which will assist in understanding the consequences of Cu incorporation on Mn_3_O_4_ thin films, and it helps to improve energy storage device performance.

## Results and Discussion

**Figure 1:**
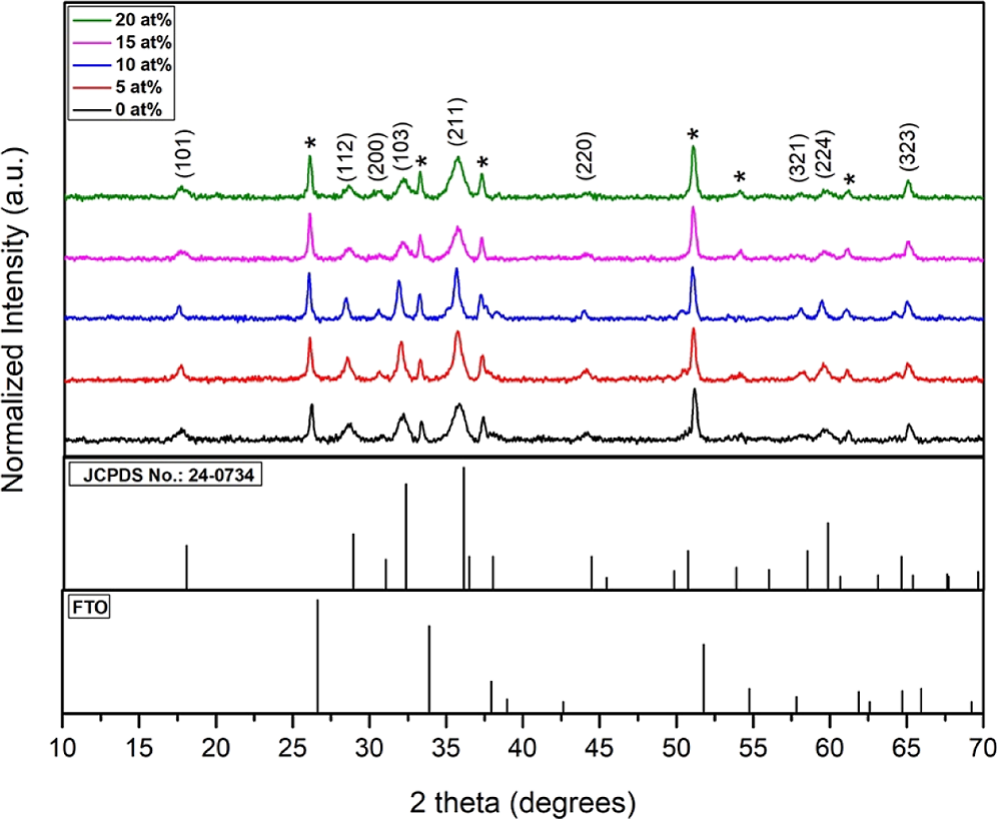
XRD pattern of Mn_3_O_4_ films with different atomic percentages of copper addition.

The thickness of the prepared electrodes were estimated using the weight difference method, and they are noticed to be in the range of 1.33–1.39 μm (mass loading of 0.65–0.68 mg/cm^2^). The crystal structure of the thin films was investigated using X-ray diffraction (XRD) and Figure 1 illustrates the XRD pattern of pristine and Cu-incorporated Mn_3_O_4_ thin film within 2θ range of 10–70°. All the patterns were matched with standard JCPDS data for both spinel Mn_3_O_4_ and a fluorine-doped tin oxide (FTO) glass substrate. The peaks corresponding to the FTO substrate were noticed due to the higher penetration depth of the XRD measurement. No additional peaks were found in the diffractogram, which indicates the formation of hausmannite Mn_3_O_4_ in the tetragonal crystal structure. For all the samples, the preferred crystal orientation was observed to be along the (211) plane and it was used to assess the crystallite size. Scherer’s relation^34^ was employed to estimate the crystallite using the following equation.
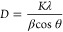
1

In this equation, β represents the peak broadening (full width at half-maximum) in radians, θ denotes the peak position in radians, λ stands for the wavelength of Cu Kα radiation employed for diffraction (λ = 1.5405 Å), and K is the shape factor with a value of 0.9. The dislocation density as well as microstrain of the films were calculated using the following relation^26,35,36^
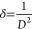
2
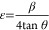
3

Estimated crystallite size, microstrain, and dislocation density are given in Table 1. Pristine Mn_3_O_4_ thin films showed a crystallite size of 8.1 nm, and it was observed to be increased to 22.1 nm corresponding to the addition of 10 at % copper. The inclusion of optimal copper dopant might facilitate grain growth, which leads to the enhancement of crystallinity. Furthermore, the addition of copper to 15 and 20 at % resulted to the reduction of crystallite size to 10.2 and 8.2 nm. The aforementioned reduction might arise due to developed strain corresponding to the incorporation of excess copper.^37^ Accordingly, the dislocation density and microstrains were varied with respect to the crystallite size. Lower dislocation density and microstrain were observed at 10 at. % of Cu-incorporation. This might be due to the higher crystallite size which may lead to a reduction in the appearance of grain boundaries.^38,39^ Besides, the computed lattice parameters and lattice spacing were quite compatible with the standard values (*a* = *b* = 5.76 Å, *c* = 9.46 Å, *d*_hkl_ = 2.77 Å). Furthermore, we found that the lattice constant was not affected significantly due to Cu incorporation.

**Table 1:** Calculated Crystallite Size, Dislocation Density, and Microstrain for Thin Films with Different Atomic Percentages of Copper Addition

sample	*d*_hkl_ (Å)	crystallite size, *D* (nm)	dislocation density, δ ×10^16^ (m^–2^)	microstrain, ε (×10^–3^)
CuM-0	2.51	8.1	1.16	12.14
CuM-5	2.51	13.9	0.51	8.11
CuM-10	2.52	22.1	0.20	5.14
CuM-15	2.51	10.2	0.96	11.09
CuM-20	2.52	8.2	1.47	13.70

**Figure 2:**
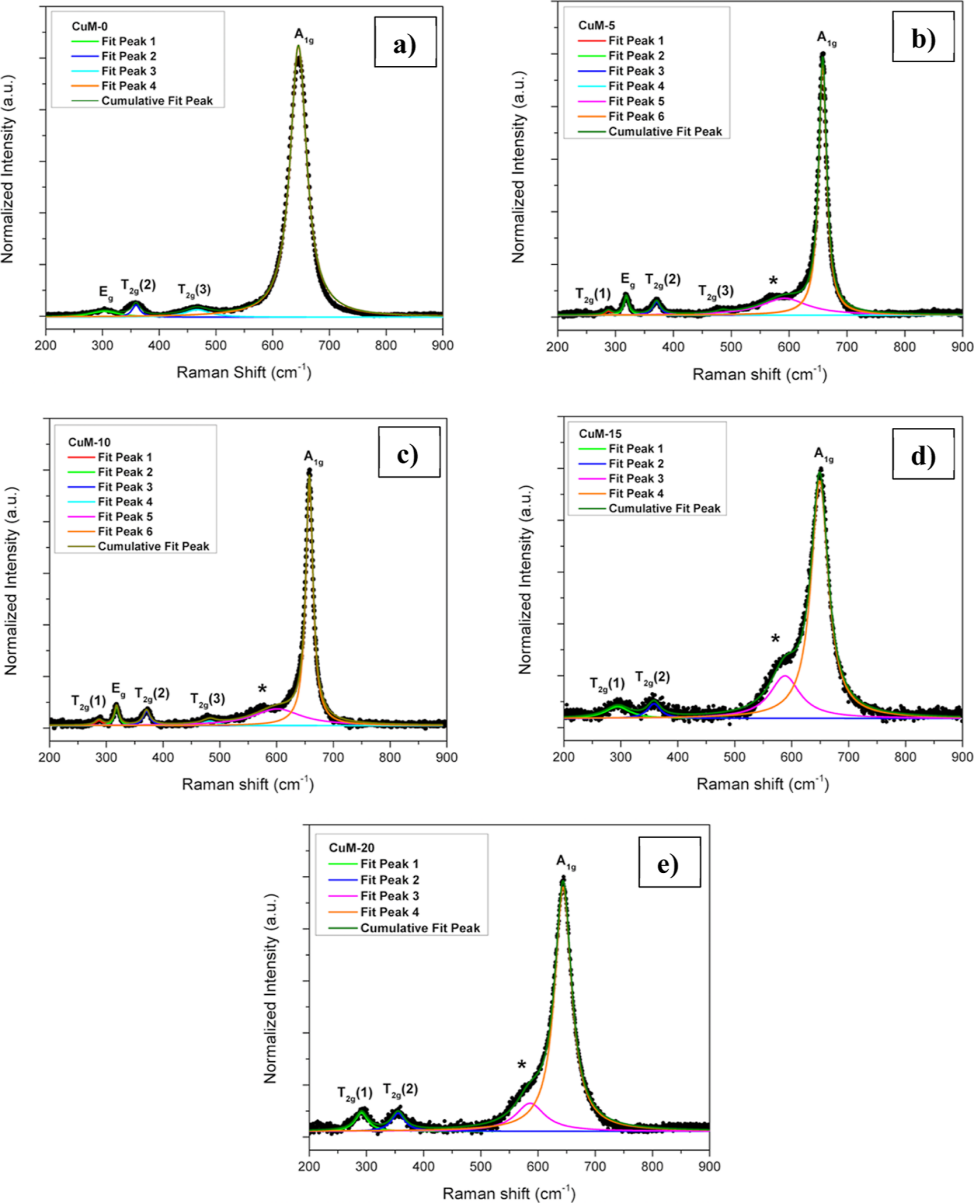
Raman spectrum of Mn_3_O_4_ films with different atomic percentages of copper addition: (a) CuM-0, (b) CuM-5, (c) CuM-10, (d) CuM-15, and (e) CuM-20.

Raman spectroscopic measurements have been carried out to obtain additional structural-related information. Figure 2 depicts the Raman spectra of pristine and Cu-incorporated Mn_3_O_4_ thin films at an excitation wavelength of 532 nm. For the pristine sample, all the observed vibrational modes were consistent with the earlier reports.^40^ The recorded peaks at 650, 316, and 361 cm^–1^ are in good accordance with the spinel Mn_3_O_4_ structure as reported in our previous work.^41^ Furthermore, lower intense peaks at around 290 cm^–1^ [*T*_2g_(1) mode] and 480 cm^–1^ [*T*_2g_(3) mode] which appeared after Cu incorporation were also characteristic vibrations of Mn_3_O_4_.^42^ Compared to the undoped film, in all doped films, a peak was observed around 590 cm^–1^, which corresponds to the stretching vibration of the Mn–O bond in Mn_2_O_3_.^12^ This broad peak suggests the presence of a small amount of Mn_2_O_3_ in the sample that arises after copper is incorporated into Mn_3_O_4_. When copper is doped into Mn_3_O_4_ it might oxidize some of the Mn^2+^ ions from Mn_3_O_4_ to Mn^3+^, causing the formation of Mn_2_O_3_. Since this formation is only in small amounts and is restricted to the surface of the films, we were unable to tackle this in XRD studies. Moreover, the observed broadening of the A_1g_ mode, where fwhm drops for 5 and 10 at. % Cu incorporated samples may be caused by the increased crystallite size as noticed in XRD. Also, the Raman modes were shifted slightly to the higher wavenumber side after Cu incorporation suggesting that Cu induces compressive strain in the samples.^43^

**Figure 3:**
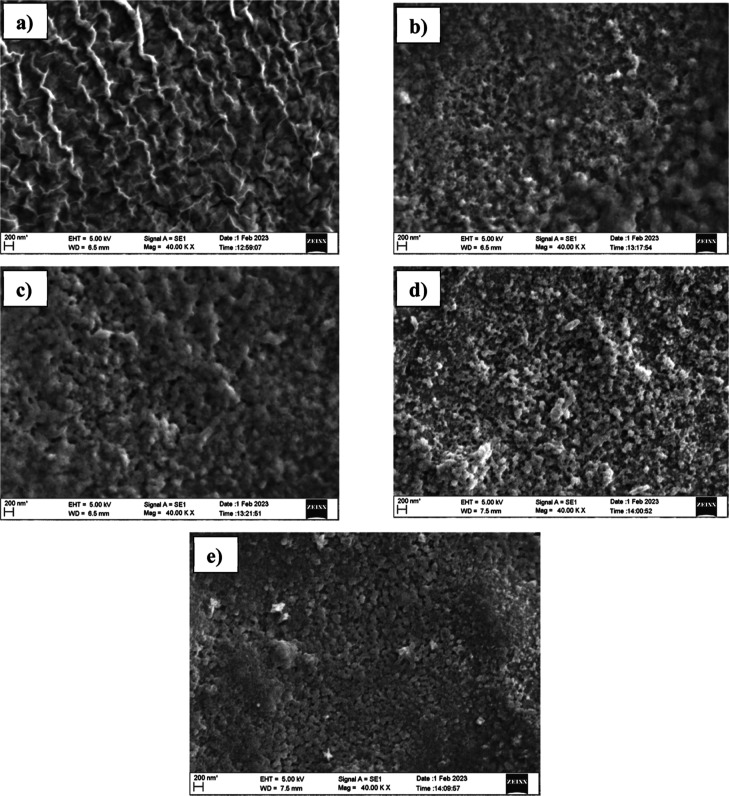
SEM images of Mn_3_O_4_ films with different atomic percentages of copper addition: (a) CuM-0, (b) CuM-5, (c) CuM-10, (d) CuM-15, and (e) CuM-20.

Figure 3 shows the scanning electron microscopy (SEM) images of pristine and copper-incorporated Mn_3_O_4_ thin films at a magnification of 40 KX. From the images, it is clear that the Cu addition has significantly changed the morphology of films. The pristine Mn_3_O_4_ film showed a fiber-like structure on its surface. Concurrently, the fiber structures disappeared in all copper-incorporated thin films. Furthermore, copper incorporation resulted in the agglomeration of particles and films become more compact. The visualized changes in the surface morphology might occur owing to the formation of the Mn_2_O_3_ phase near the surface.

The chemical composition of pristine and copper-incorporated Mn_3_O_4_ thin films was investigated by using energy-dispersive spectroscopy (EDS). Figure S2 provides the EDS spectra of all of the synthesized films. All the films showed near stoichiometry composition ratio.^44^Table 2 presents the calculated elemental atomic percentage of Mn, O, and Cu ions in thin films. The pristine Mn_3_O_4_ thin films show the 33.15% percentage of the Mn element. The significant reduction in Mn at. % was noticed corresponding to the increase of copper addition. For the films deposited using higher at. % copper, i.e., 20 shows the reduction in Mn at. % to 20.8. Furthermore, considerable enhancement in the Cu atomic percentage was noticed in thin films corresponding to the addition of Cu content in the precursor solution, which implies the successful incorporation of Cu ions on the deposited Mn_3_O_4_ thin films.

**Table 2:** Composition of Mn_3_O_4_ Films with Different Atomic Percentages of Copper Addition

	elements		
Sample	Mn (at. %)	O (at. %)	Cu (at. %)
CuM-0	33.15	66.85	0
CuM-5	34.08	63.72	2.19
CuM-10	29.56	67.42	3.02
CuM-15	24.75	67.23	8.01
CuM-20	20.84	66.87	12.29

**Figure 4:**
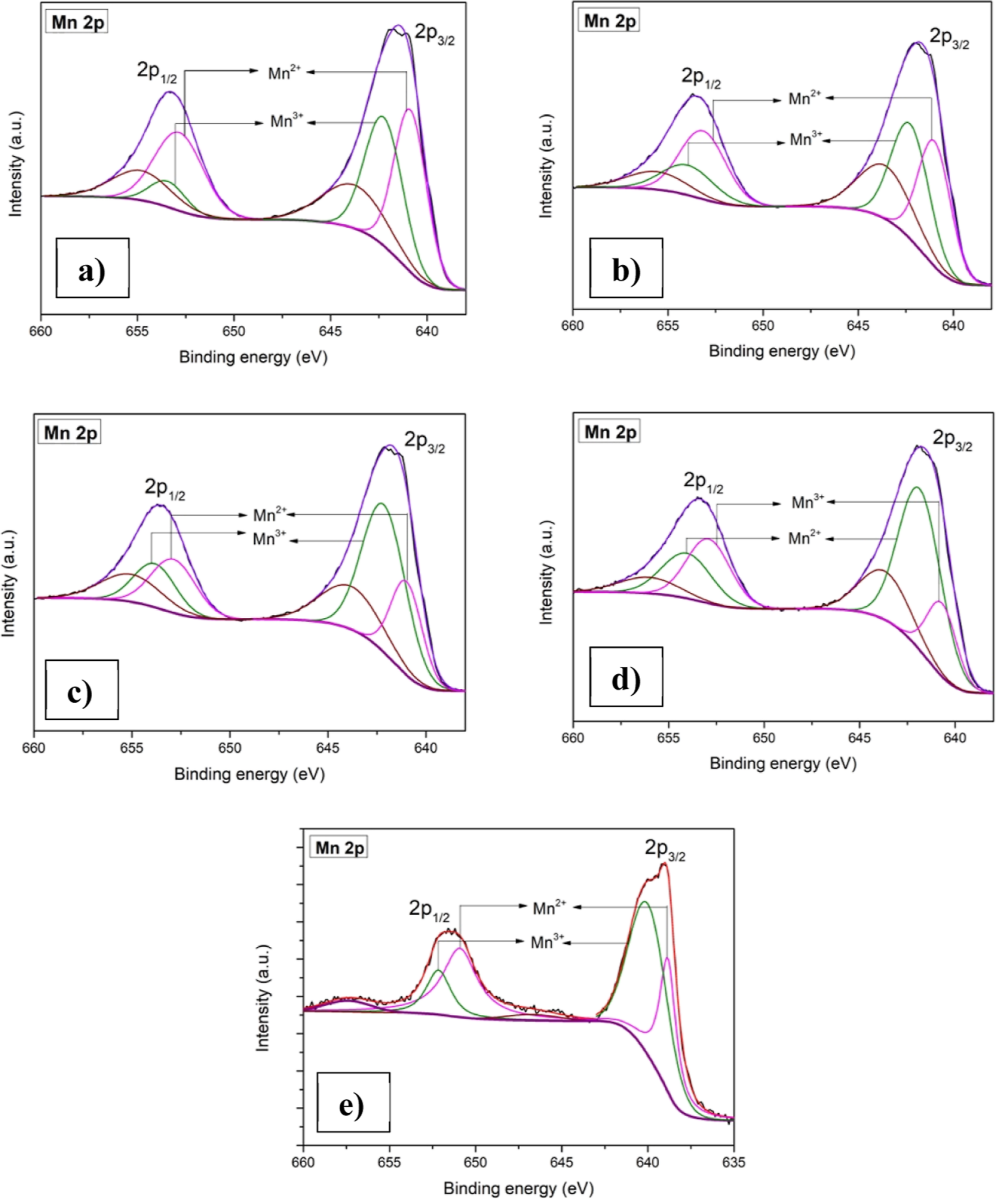
XPS core spectra of Mn 2p: (a) CuM-0, (b) CuM-5, (c) CuM-10, (d) CuM-15, and (e) CuM-20.

The details about the oxidation state were obtained by X-ray photoelectron spectroscopy (XPS). Figure S1 gives the survey spectra of all of the synthesized thin films. Figure 4 illustrates the Mn 2p core spectra of pristine and copper-incorporated Mn_3_O_4_ thin films. The deconvolution of the spectra, which has been detailed in our previous work,^41^ reveals the presence of mixed valency, i.e., Mn^2+^ and Mn^3+^. Furthermore, if we compare the area under each peak, it was observed that as the copper has been incorporated, the Mn^3+^ state was seen to be increasing, which indicates the oxidation process. This increase in the Mn^3+^/Mn^2+^ ratio indicates the formation of the Mn_2_O_3_ phase, as observed in Raman studies.

**Figure 5:**
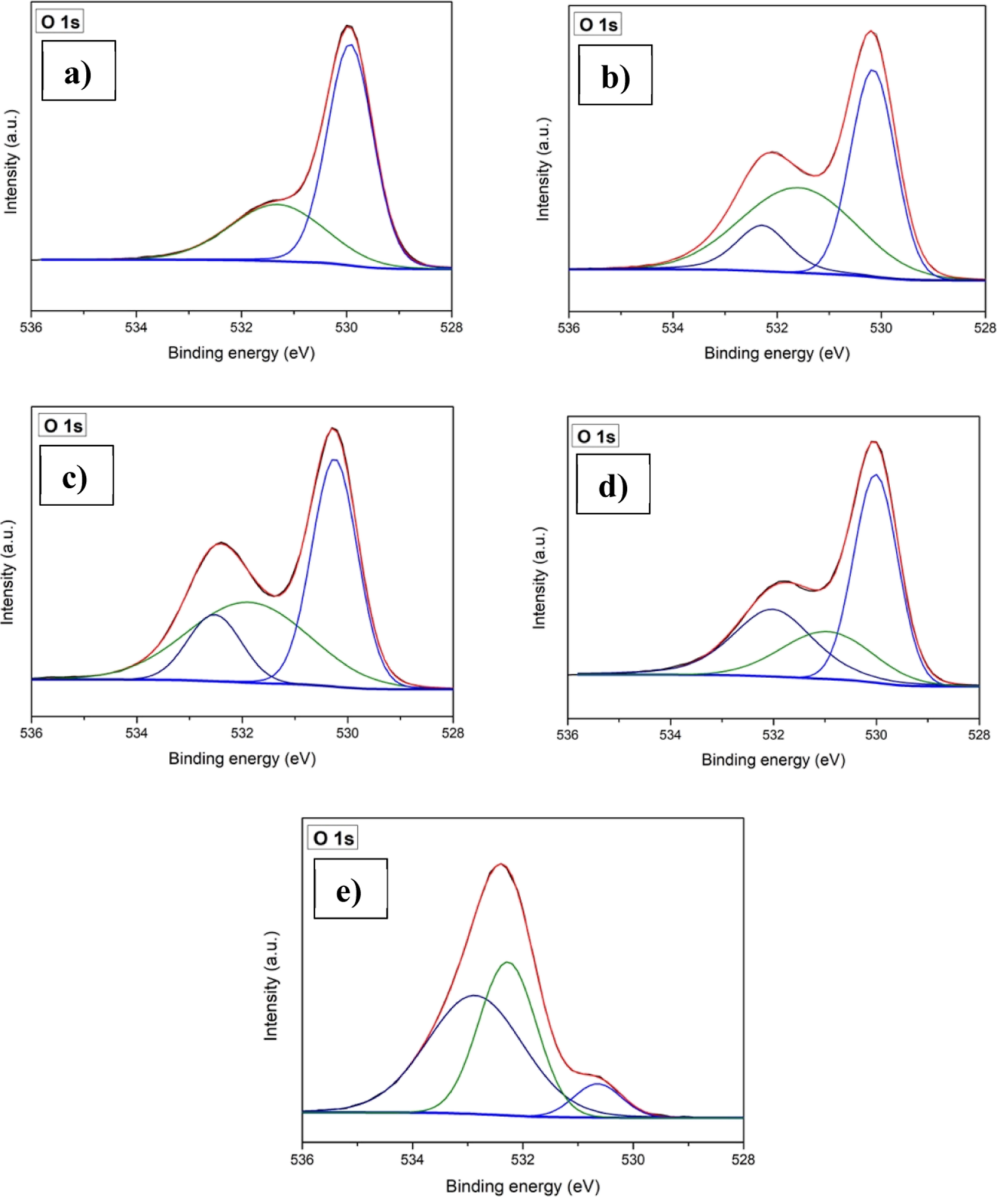
XPS core spectra of the O 1s: (a) CuM-0, (b) CuM-5, (c) CuM-10, (d) CuM-15, and (e) CuM-20.

In Figure 5, the O 1s core spectra are presented for both the pristine Mn_3_O_4_ thin film and the Cu-incorporated Mn_3_O_4_ thin films. In the pristine film, the observed two distinct peaks at 529.9 and 531.3 eV, attributes to the binding energies associated with the metal–oxygen bonds within the lattice (O_lat_) and the presence of oxygen defects or vacancies (O_vac_), respectively.^45^ All the copper-incorporated samples exhibited one extra peak at around 532 eV which may be occurred due to the surface-adsorbed H_2_O/O_2_ (O_abs_).^31^ Moreover, upon Cu- incorporation there was variations in the observed peak intensities of O_lat_, O_vac_, as well as O_abs_ which clearly indicates the changes in the chemical state of the oxygen near the surface. Upon Cu-incorporation, the relative area of O_lat_ decreases with respect to the O_vac_ along with some fluctuations caused by O_abs_.^46^ These findings imply that Cu-incorporation increases the number of defects or oxygen vacancies in the samples which could be beneficial for the electrochemical reaction.^47^

**Figure 6:**
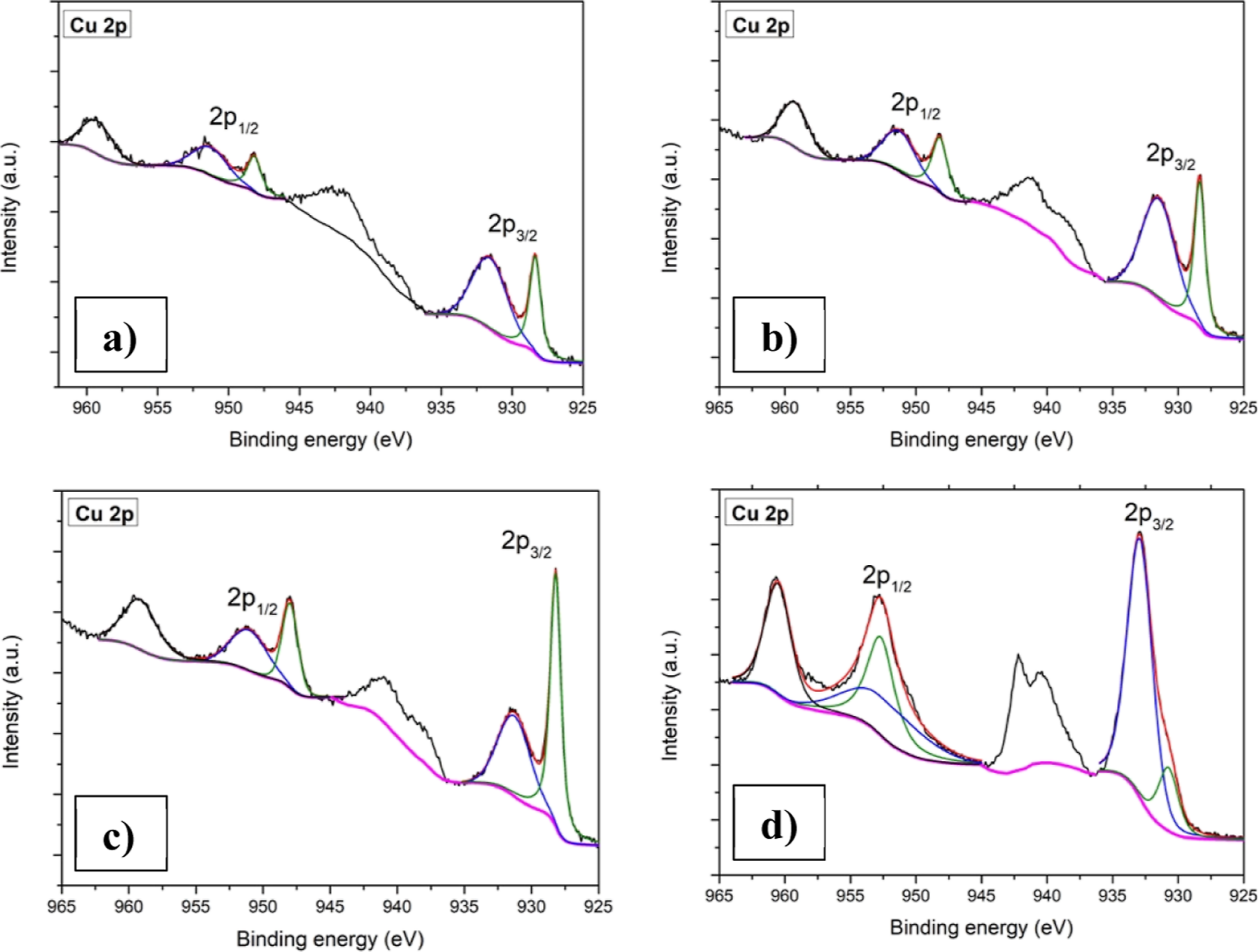
XPS core spectra of Cu 2p: (a) CuM-5, (b) CuM-10, (c) CuM-15, and (d) CuM-20.

Figure 6 displays the Cu 2p core spectra for all of the Cu-incorporated samples. All the samples showed two peaks around 929–932 and 949–953 eV corresponding to 2p_3/2_ and 2p_1/2_ along with their respective satellite peaks. Each of the two peaks was deconvoluted into two more peaks which can be assigned to the Cu^+^ and Cu^2+^ state.^48^

**Figure 7:**
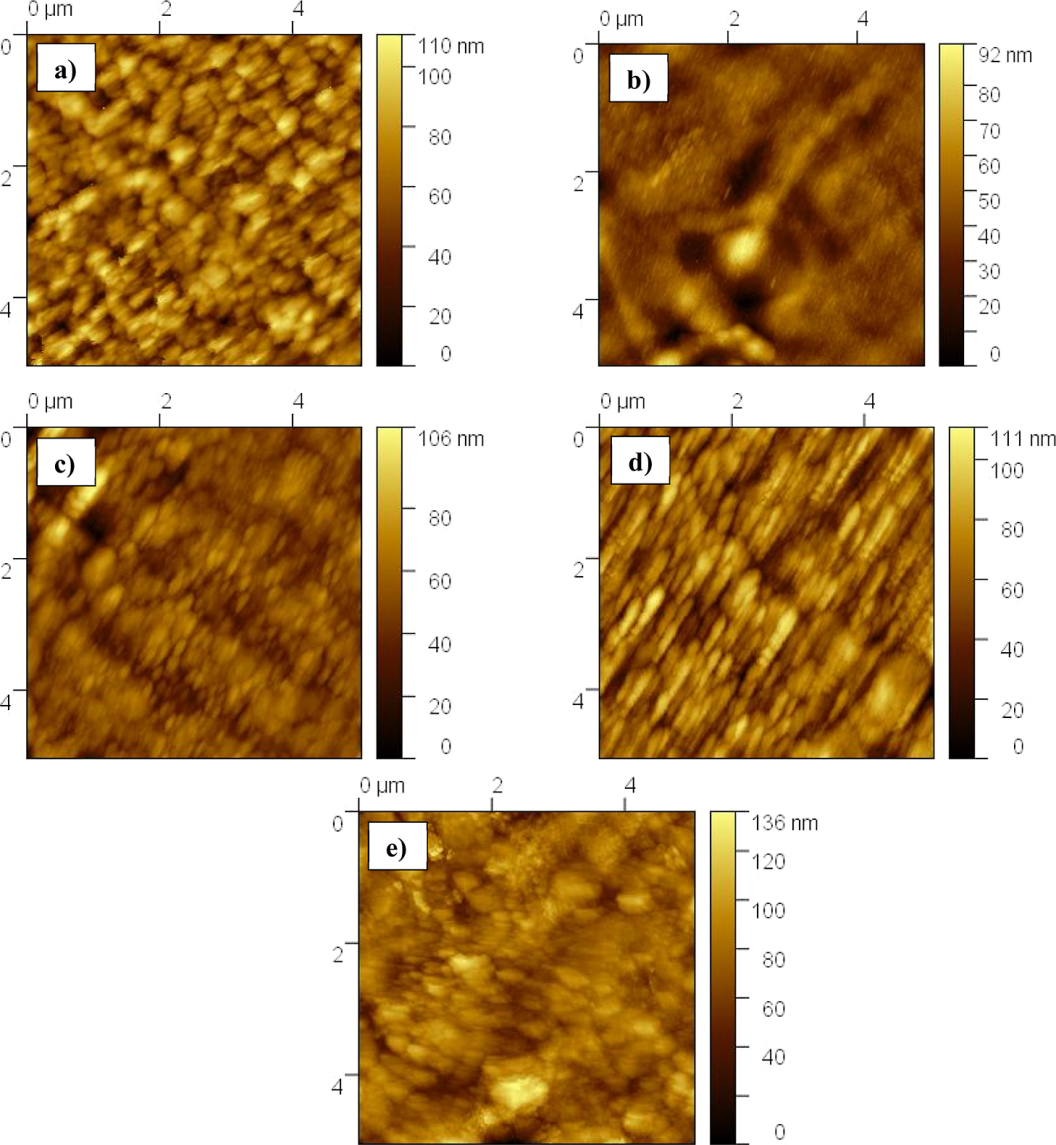
AFM images of Mn_3_O_4_ films with different atomic percentages of copper addition, (a) CuM-0, (b) CuM-5, (c) CuM-10, (d) CuM-15, and (e) CuM-20.

Figure 7 illustrates topographic atomic force microscopy (AFM) images of pristine and Cu- incorporated Mn_3_O_4_ thin films, and computed mean roughness (*R*_a_) as well as root-mean-square roughness (*R*_q_) are given in Table 3. The pristine Mn_3_O_4_ thin films exhibited a *R*_q_ value of 15.3 nm. In that, the addition of 5 at. % of Cu resulted in the minimization of the *R*_q_ value to 10.7 nm. Furthermore, an increase of Cu content, i.e., 10 at. %, leads to the lesser *R*_q_ of 10.1 nm. The observed smoothness in the film might be ascribed to its demonstrated higher crystallinity on the XRD pattern. Beyond this, the higher Cu incorporation of 15 and 20 at. % shows the higher *R*_q_ values of 14.3 and 14.9 nm, respectively. However, the less roughness films were not preferred for electrochemical applications due to their less surface area, which limits the electroactive sites.^49^

**Table 3:** Surface Roughness Parameters for Pristine and Copper-Incorporated Mn_3_O_4_ Films

sample	*R*_a_ (nm)	*R*_q_ (nm)
CuM-0	12.3	15.3
CuM-5	7.9	10.7
CuM-10	7.2	10.1
CuM-15	11.1	14.3
CuM-20	11.9	14.9

**Figure 8:**
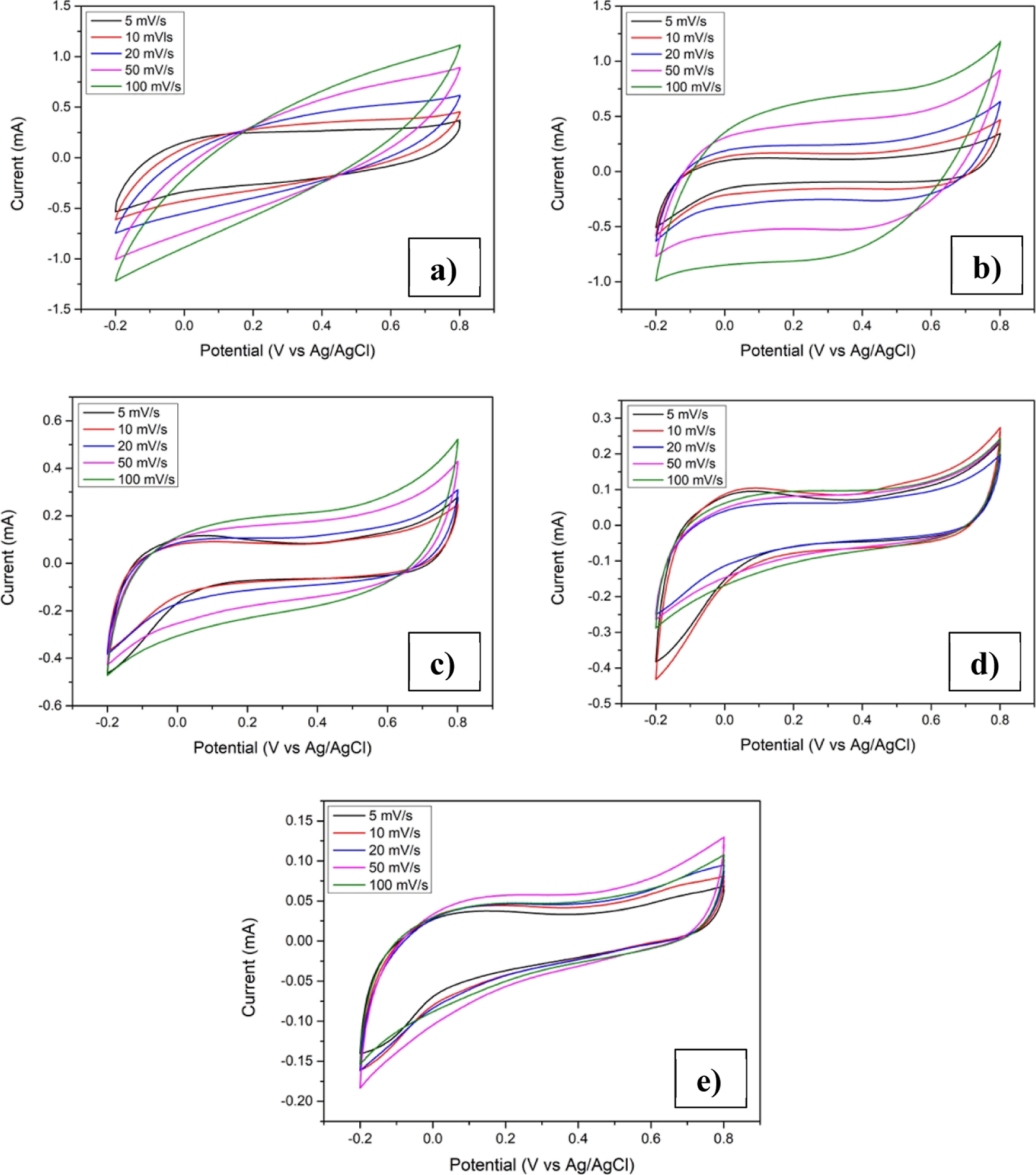
CV of Mn_3_O_4_ films with different atomic percentages of copper addition: (a) CuM-0, (b) CuM-5, (c) CuM-10, (d) CuM-15, and (e) CuM-20.

Figure 8 depicts the cyclic voltammogram (CV) of the pristine and Cu-incorporated Mn_3_O_4_ thin film electrodes. All the CV plots showed a divergence from the rectangular shape, which reveals the pseudocapacitive nature. Even after copper was introduced, the pseudocapacitor behavior was still retained in all the electrodes.

The specific capacitance was calculated using the CV plot utilizing the relation^50^
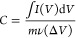
4where ∫*I*(*V*)d*V* provides the area under the CV curve, “*m*” implies the mass deposited, “ν” denotes the scan rate, whereas “Δ*V*” indicates the applied potential window.

The specific capacitances calculated were found to be 132, 73, 64, 53, and 22 F/g for CuM-0, CuM-5, CuM-10, CuM-15, and CuM-20 samples, respectively, at 5 mV/s. The addition of Cu resulted in a reduction of capacitance value in comparison to the pristine sample. The aforementioned result indicates that Cu incorporation did not assist to augment the performance of the Mn_3_O_4_ electrodes. This decrement in specific capacitance could be attributed to the formation of the Mn_2_O_3_ secondary phase as seen in Raman studies for Cu-incorporated Mn_3_O_4_ thin films. According to previous reports, the Mn_2_O_3_ phase can suppress the electrochemical performance of Mn_3_O_4_ because of its relatively lower conductivity as compared to Mn_3_O_4_.^40,51^ In addition to that, the Mn_2_O_3_ phase formed at the surface may act as a passivating layer or barrier which can inhibit the electrochemical reactions taking place at the electrode/electrolyte interface. Thus, the formation of mixed manganese oxide phases on Cu incorporation resulted in the reduction in the specific capacitance of the electrode.

**Figure 9:**
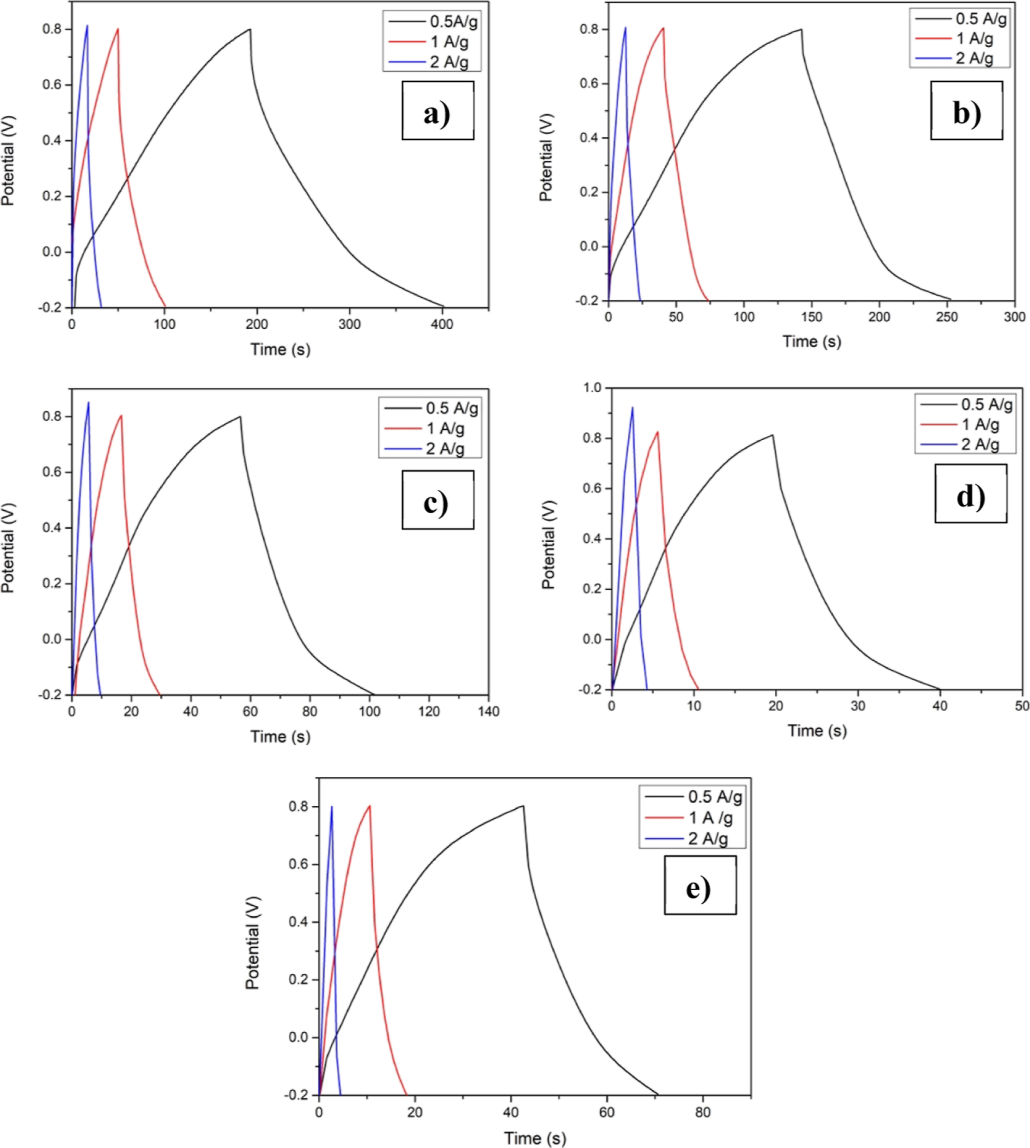
GCD plots of Mn_3_O_4_ films with different atomic percentages of copper addition: (a) CuM-0, (b) CuM-5, (c) CuM-10, (d) CuM-15, and (e) CuM-20.

Figure 9 shows the galvanostatic charge–discharge (GCD) curves of pristine and copper-incorporated Mn_3_O_4_ thin films recorded at different current densities. Again, here the almost symmetric nature of the curve shows the pseudocapacitive behavior of the electrodes. Similar to CV studies, here also specific capacitance showed a decrement as a result of Cu incorporation. The specific capacitance was computed using the GCD plot, making use of the relation^52^
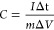
5where “*I*” implies the discharge current, “*m*” denotes the mass deposited, “Δt” gives the discharge time, and “Δ*V*” indicates the applied potential window. The specific capacitance values are found to be 112, 60, 24, 16, and 12 F/g at 0.5 A/g for CuM-0, CuM-5, CuM-10, CuM-15, and CuM-20, respectively. Furthermore, a drop in specific capacitance is seen with a rise in current density, which may be owing to the insufficient time for the appropriate reactions to occur at higher current densities.^53^ However, the obtained specific capacitance for the pristine sample is higher than the earlier reported values.^54,55^

**Figure 10:**
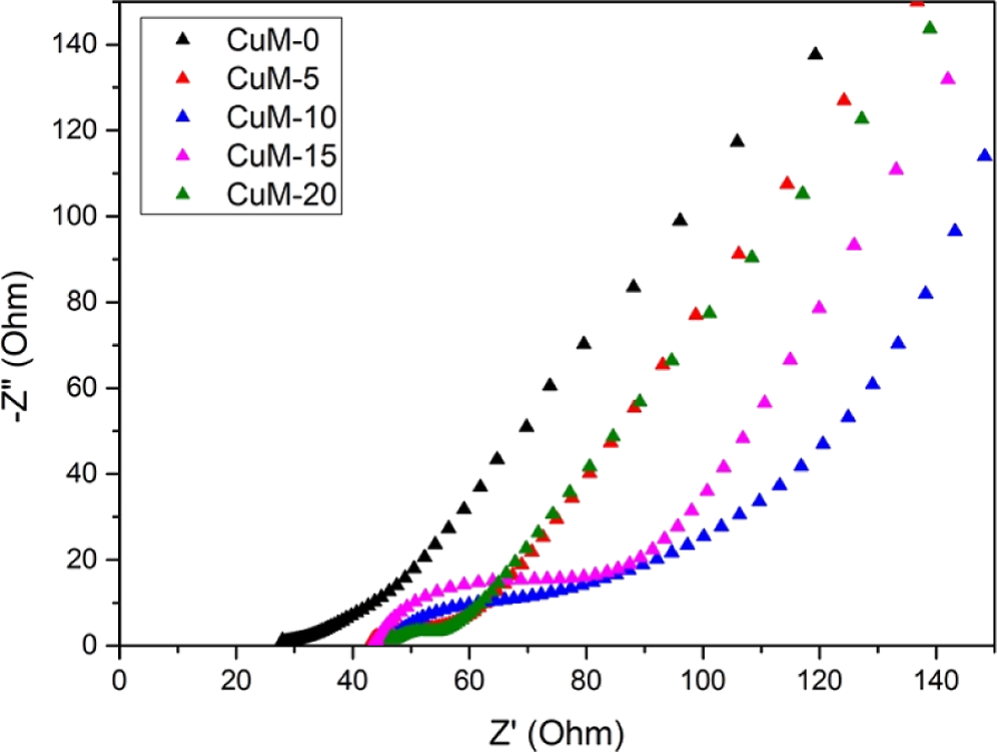
Nyquist plots of Mn_3_O_4_ films with different atomic percentages of copper addition.

Figure 10 shows the Nyquist plots for the pristine and copper-incorporated Mn_3_O_4_ thin films. The decrement in the specific capacitance as seen from CV and GCD can further be supported by EIS results. Here, we can observe that after Cu incorporation the charge transfer resistance as well as series resistance (which can be obtained from the semicircle in the midfrequency region and x-intercept at high frequency region) were increased because of which the specific capacitance got reduced. This increment in charge transfer resistance could be ascribed to the increase in crystallite size, as noticed from XRD. However, the lower conductivity of the Mn_2_O_3_ phase may also contribute to the increase in series resistance. Furthermore, it was observed that charge transfer resistance tends to decrease after 10 at. %, which may be attributed to the decrement in the crystallite size as well as an increase in the structural water component in the samples as observed by XPS O 1s core spectra and similar results were obtained in previous reports.^56^ Even though the R_ct_ has improved, we have not noticed any changes in the specific capacitance. This can be due to the higher resistance offered by the Mn_2_O_3_ phase. Thus, in order to perform better, a proper balance between conductivity and capacitance is absolutely necessary.^57^

**Figure 11:**
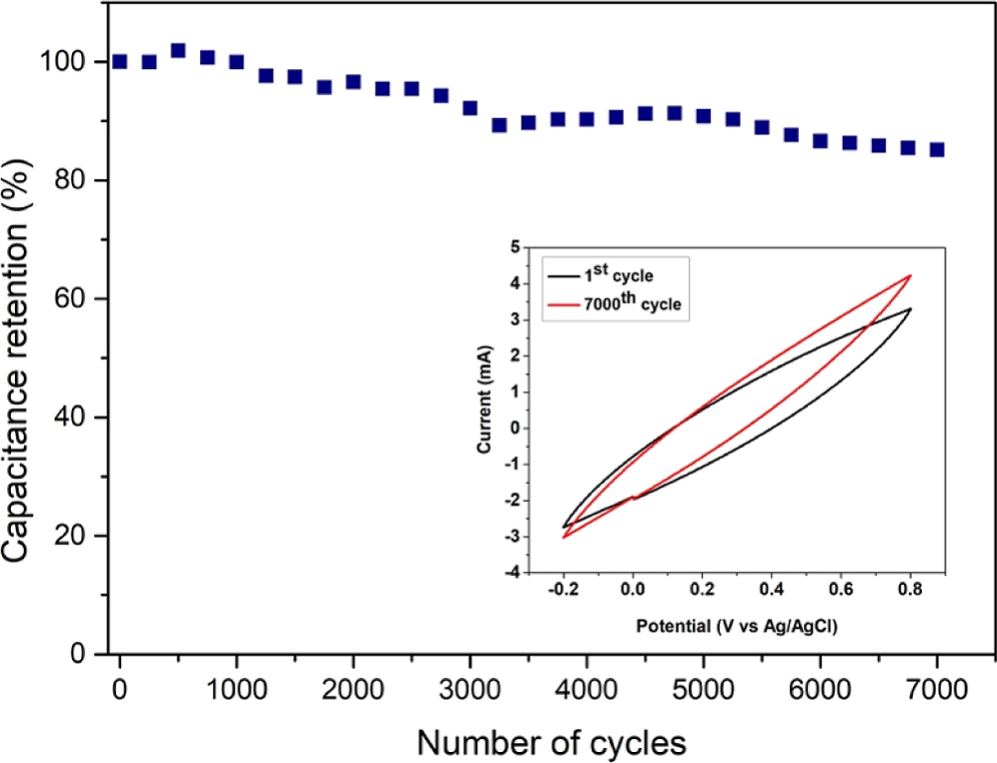
Capacitance retention of pristine Mn_3_O_4_ electrode at a scan rate of 500 mV/s.

Additionally, the long-term stability test of the pristine Mn_3_O_4_ has been performed using the CV configuration in the same setup at a scan rate of 500 mV/s. Figure 11 shows the stability test results with an inset showing comparison between the first and 7000th cycle. The electrode retain 85% of its initial capacitance even after 7000 cycles. This shows as tremendous electrochemical stability of the prepared electrodes compared to earlier reports.^58,59^ Furthermore, the observed small increase and decrease in the capacitance gives an indication of ion diffusion mechanism happening at the electrode.^60^

## Conclusions

In the current work, the consequences of copper incorporation on the electrochemical performance of Mn_3_O_4_ thin films have been examined. Cu has been incorporated in Mn_3_O_4_ at different atomic percentages, i.e., 5, 10, 15, and 20 at. %. XRD data demonstrated the formation of a tetragonal crystal structure. The thin film with 10 at. % of Cu exhibited a higher crystallinity in comparison with other samples. Furthermore, Raman studies confirmed the formation of the Mn_2_O_3_ secondary phase for all the Cu-incorporated Mn_3_O_4_ thin films. This was again supported by XPS, which showed an increase in the Mn^3+^/Mn^2+^ ratio. Surface micrographs displayed that Cu incorporation had significantly affected the morphology and EDS results confirmed the presence of Mn, O, and Cu in the prepared samples. Additionally, AFM results demonstrated that copper addition up to 10 at. % had smoothed the film surface, and thereafter roughness increased. Moreover, using 0.1 M Na_2_SO_4_ as the electrolyte, electrochemical tests were made to determine the capacitance performance in three electrode systems. CV studies displayed a reduction in the specific capacitance after Cu incorporation into Mn_3_O_4_ due to the formation of Mn_2_O_3_. A similar trend has been observed in GCD outcomes, as well. EIS studies also demonstrated the impact of Cu incorporation on variation of charge transfer resistance. Compared to Cu-incorporated samples, pristine Mn_3_O_4_ showed better performance with a maximum specific capacitance of up to 132 F/g. These findings offer insightful information about the effects of Cu incorporation in the Mn_3_O_4_ thin-film electrode properties. Furthermore, the attained results pave the way to effectively improving the performance of Mn_3_O_4_ thin films for energy storage applications.

## Experimental Procedure

Thin-film preparation: Mn_3_O_4_ thin films were synthesized via a chemical spray pyrolysis technique on fluorine-doped tin oxide (FTO)-coated glass substrates. The cleaning procedure has been detailed in our previous work.^41^ The precursor solution was prepared by dissolving manganese acetate tetrahydrate [Mn(CH_3_OO)_2_·4H_2_O] in double distilled water. Optimized molar concentration of 0.06 M was employed in this present study. The copper chloride dihydrate (CuCl_2_·2H_2_O) solution was used as a source of copper. The Cu/Mn ratio was maintained at 5, 10, 15, and 20 at. % in the precursor solution. The precursors were stirred with the help of a magnetic stirrer at room temperature for a duration of 1 h to get a homogeneous solution. The substrate temperature was set to 325 °C, which had been optimized for the process. Furthermore, compressed air was utilized as the carrier gas at a pressure of 1 bar, and the distance between the nozzle to the substrate was kept at 14 cm.

Possible mechanism for film formation: Mn_3_O_4_ thin films were formed by the pyrolytic decomposition of a [Mn(CH_3_OO)_2_·4H_2_O] solution. The initial precursor solution will provide Mn^2+^ ions which will react with 2OH^–^ in the solution to form Mn(OH)_2_. This will then decompose to MnO and finally at the optimized temperature, it will be oxidized to form Mn_3_O_4_. The overall reaction can be written as follows^61^
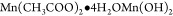
6
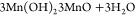
7
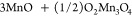
8

Furthermore, the addition of (CuCl_2_·2H_2_O) will provide Cu^2+^ which will partially oxidize Mn_3_O_4_ to Mn_2_O_3_
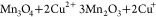
9

Thin film characterization: the structural details of all the samples were collected via an XRD pattern using a Rigaku Miniflex 600. The Cu Kα (λ = 1.5405 Å) radiation was used, and the scanning was performed in the range of 10 to 70°. Further LabRAM (excitation wavelength-532 nm; objective-×100; accumulation time-15 s; grating-1800, laser power: 0.836 mW) Raman spectroscopy was employed to record the Raman vibrational modes. The XPS studies were done by utilizing Axis Ultra and the obtained spectra were calibrated using C 1s spectra. The surface morphology as well as elemental composition of the samples were analyzed through SEM and EDS employing Zeiss Sigma and Oxford instruments (with an acceleration voltage of 15 kV), respectively. Surface topography was studied by AFM employing Flex-Axiom AFM. The topographs were processed, and roughness parameters were estimated using Gwyddion analysis software (version 2.61).

### Electrochemical Measurements

The electrochemical behavior of the synthesized thin-film electrodes was performed in a three-electrode system (Metrohm, Autolab PGSTAT204). The Ag/AgCl and Pt were employed as the reference electrode and the counter electrode, respectively. The as-synthesized thin film samples, which had a dimension of 1 cm^2^ × 1 cm^2^ as the active area, were utilized as working electrodes. As an electrolyte, a 0.1 M Na_2_SO_4_ solution was employed. The cyclic voltammetry (CV) studies were carried out in the potential range of −0.2 to 0.8 V versus Ag/AgCl at scan rates of 5, 10, 20, 50, and 100 mV/s. Furthermore, galvanostatic charge/discharge (GCD) analyses were conducted in the same voltage window at current densities of 0.5, 1, and 2 A/g. The electrochemical impedance spectra (EIS) were recorded for a frequency range from 10 mHz to 1 MHz. All the measurements were done at ambient temperature.

The electrochemical reactions taking place at the Mn_3_O_4_ electrode in Na_2_SO_4_ electrolyte can be written as^51^
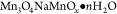

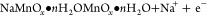


This reaction is due to the adsorption of solvated ions in the electrolyte on the electrode surface.

## Supporting Information Available

XPS survey spectra and EDS spectra of all the samples (PDF)

## References

[ref1] Bigdeloo M.; Kowsari E.; Ehsani A.; Chinnappan A.; Ramakrishna S.; AliAkbari R. Review on Innovative Sustainable Nanomaterials to Enhance the Performance of Supercapacitors. J. Energy Storage 2021, 37, 102474 10.1016/j.est.2021.102474.

[ref2] Mascarenhas F. J.; Hegde S. S.; Bhat B. R. Supercapattery. Sustainable Materials for Electrochemical Capacitors; John Wiley & Sons, Ltd, 2023; pp 279–290.

[ref3] Abdel Maksoud M. I. A.; Fahim R. A.; Shalan A. E.; Abd Elkodous M.; Olojede S. O.; Osman A. I.; Farrell C.; Al-Muhtaseb A. H.; Awed A. S.; Ashour A. H.; Rooney D. W. Advanced Materials and Technologies for Supercapacitors Used in Energy Conversion and Storage: A Review, Environ. Chem. Lett., Springer International Publishing, 2020; 19, pp 375-439. 10.1007/s10311-020-01075-w

[ref4] Hegde S. S.; Bhat B. R. Solid Waste-Derived Carbon Materials for Electrochemical Capacitors. Sustainable Materials for Electrochemical Capacitors; John Wiley & Sons, Ltd, 2023; pp 19–31.

[ref5] Yuan S.; Duan X.; Liu J.; Ye Y.; Lv F.; Liu T.; Wang Q.; Zhang X. Recent Progress on Transition Metal Oxides as Advanced Materials for Energy Conversion and Storage. Energy Storage Mater. 2021, 42, 317–369. 10.1016/j.ensm.2021.07.007.

[ref6] Amara M. A.; Larbi T.; Labidi A.; Karyaoui M.; Ouni B.; Amlouk M. Microstructural, Optical and Ethanol Sensing Properties of Sprayed Li-Doped Mn_3_O_4_ Thin Films. Mater. Res. Bull. 2016, 75, 217–223. 10.1016/j.materresbull.2015.11.042.

[ref7] Xiong S.; Peng Y.; Wang D.; Huang N.; Zhang Q.; Yang S.; Chen J.; Li J. The Role of the Cu Dopant on a Mn_3_O_4_ Spinel SCR Catalyst: Improvement of Low-Temperature Activity and Sulfur Resistance. Chem. Eng. J. 2020, 387, 124090 10.1016/j.cej.2020.124090.

[ref8] Bischoff C.; Fitz O.; Schiller C.; Gentischer H.; Biro D.; Henning H. M. Investigating the Impact of Particle Size on the Performance and Internal Resistance of Aqueous Zinc Ion Batteries with a Manganese Sesquioxide Cathode. Batteries 2018, 4 (3), 44 10.3390/batteries4030044.

[ref9] Sayyed S. G.; Shaikh A. V.; Dubal D. P.; Pathan H. M. Paving the Way towards Mn_3_O_4_ Based Energy Storage Systems. ES Energy Environ. 2021, 3–21. 10.30919/esee8c522.

[ref10] Sakib M. N.; Ahmed S.; Rahat S. M. S. M.; Shuchi S. B. A Review of Recent Advances in Manganese-Based Supercapacitors. J. Energy Storage 2021, 44, 103322 10.1016/j.est.2021.103322.

[ref11] Sriram S. R.; Parne S. R.; Pothukanuri N.; Joshi D.; Edla D. R. Facile Synthesis of Pure and Cr-Doped WO_3_ Thin Films for the Detection of Xylene at Room Temperature. ACS Omega 2022, 7 (51), 47796–47805. 10.1021/acsomega.2c05589. 36591164 PMC9798732

[ref12] Radinger H.; Connor P.; Stark R.; Jaegermann W.; Kaiser B. Manganese Oxide as an Inorganic Catalyst for the Oxygen Evolution Reaction Studied by X-Ray Photoelectron and Operando Raman Spectroscopy. ChemCatChem 2021, 13 (4), 1175–1185. 10.1002/cctc.202001756.

[ref13] Sayyed S. G.; Pathan H. M.; Shaikh A. V.; Shaikh S. F.; Al-Enizi A. M. Investigation of Electrochemical Performance and Stability of Electrodeposited Mn_3_O_4_ Thin Films in Different Aqueous Electrolytes for Its Application in Flexible Supercapacitors. J. Energy Storage 2021, 33, 102076 10.1016/j.est.2020.102076.

[ref14] Nkele A. C.; Chime U.; Ezealigo B.; Nwanya A.; Agbogu A. N. C.; Ekwealor A. B. C.; Osuji R. U.; Ejikeme P. M.; Maaza M.; Ezema F. Enhanced Electrochemical Property of SILAR-Deposited Mn_3_O_4_ Thin Films Decorated on Graphene. J. Mater. Res. Technol. 2020, 9 (4), 9049–9058. 10.1016/j.jmrt.2020.06.031.

[ref15] Ambare R. C.; Bharadwaj S. R.; Lokhande B. J. Electrochemical Characterization of Mn: Co_3_O_4_ Thin Films Prepared by Spray Pyrolysis via Aqueous Route. Curr. Appl. Phys. 2014, 14 (11), 1582–1590. 10.1016/j.cap.2014.08.001.

[ref16] Ambare R. C.; Khavale S. V.; Nakate U. T.; Khanvilkar M. B.; Lokhande B. J. Electrochemical Investigations of Spray Pyrolysed Ruthenium Incorporated Co_3_O_4_ Electrodes Prepared via Aqueous Route. Colloids Surf., A 2021, 615, 126215 10.1016/j.colsurfa.2021.126215.

[ref17] Moses Ezhil Raj A.; Victoria S. G.; Jothy V. B.; Ravidhas C.; Wollschläger J.; Suendorf M.; Neumann M.; Jayachandran M.; Sanjeeviraja C. XRD and XPS Characterization of Mixed Valence Mn_3_O_4_ Hausmannite Thin Films Prepared by Chemical Spray Pyrolysis Technique. Appl. Surf. Sci. 2010, 256 (9), 2920–2926. 10.1016/j.apsusc.2009.11.051.

[ref18] Ambare R. C.; Bharadwaj S. R.; Lokhande B. J. Spray Pyrolysed Mn:Co_3_O_4_ Thin Film Electrodes via Non-Aqueous Route and Their Electrochemical Parameter Measurements. Measurement 2016, 88, 66–76. 10.1016/j.measurement.2016.02.063.

[ref19] Perednis D.; Gauckler L. J. Thin Film Deposition Using Spray Pyrolysis. J. Electroceram. 2005, 14 (2), 103–111. 10.1007/s10832-005-0870-x.

[ref20] Ambare R. C.; Lokhande B. J. Solution Concentration and Decomposition Temperature Dependent Electrochemical Behavior of Aqueous Route Spray Pyrolysed Mn_3_O_4_: Supercapacitive Approach. J. Mater. Sci.: Mater. Electron. 2017, 28 (16), 12246–12252. 10.1007/s10854-017-7040-1.

[ref21] Ambare R. C.; Lokhande B. J. Ru Incorporation Enhanced Electrochemical Performance of Spray Deposited Mn: Co_3_O_4_ Nano-Composite: Electrochemical Approach. J. Anal. Appl. Pyrolysis 2018, 132, 245–253. 10.1016/j.jaap.2018.01.013.

[ref22] Pramitha A.; Raviprakash Y. Recent Developments and Viable Approaches for High-Performance Supercapacitors Using Transition Metal-Based Electrode Materials. J. Energy Storage 2022, 49, 104120 10.1016/j.est.2022.104120.

[ref23] Ambare R. C.; Lokhande B. J. Spray Pyrolyzed Ni Incorporated Cobalt Oxide Thin Film Electrodes and Their Electrochemical Study. J. Mater. Sci.: Mater. Electron. 2018, 29 (19), 16289–16294. 10.1007/s10854-018-9718-4.

[ref24] Khavale S. V.; Ambare R. C.; Lokhande B. J. Molar Optimization of MnO_2_ to Form Composite with Co_3_O_4_ by Potentiodynamic Electrodeposition for Better Electrochemical Characterizations. J. Mater. Sci.: Mater. Electron. 2020, 31 (10), 7315–7323. 10.1007/s10854-019-02420-8.

[ref25] Naiknaware A. G.; Chavan J. U.; Kaldate S. H.; Yadav A. A. Studies on Spray Deposited Ni Doped Mn_3_O_4_ Electrodes for Supercapacitor Applications. J. Alloys Compd. 2019, 774, 787–794. 10.1016/j.jallcom.2018.10.001.

[ref26] Liu Z.; Sun G.; Chen C.; Sun K.; Zeng L.; Yang L.; Chen Y.; Wang W.; Liu B.; Lu Y.; Pan Y.; Liu Y.; Liu C. Fe-Doped Mn_3_O_4_Spinel Nanoparticles with Highly Exposed Fe_oct_-O-Mn_tet_ Sites for Efficient Selective Catalytic Reduction (SCR) of NO with Ammonia at Low Temperatures. ACS Catal. 2020, 10 (12), 6803–6809. 10.1021/acscatal.0c01284.

[ref27] Paulraj I.; Senguttuvan G.; Chang J.; Raj M.; Kumar N. Effect of Cr Doping on Mn_3_O_4_ Thin Films for High-Performance Supercapacitors. J. Mater. Sci.: Mater. Electron. 2021, 32, 1–11. 10.1007/s10854-020-05118-4.

[ref28] Bayram O.; İgman E.; Guney H.; Simsek O. The Role of Cobalt Doping on the Optical and Structural Properties of Mn_3_O_4_ Nanostructured Thin Films Obtained by SILAR Technique. Superlattice. Microst. 2019, 128, 212–220. 10.1016/j.spmi.2019.01.025.

[ref29] Bayram O.; Ertargin M. E.; Igman E.; Guney H.; Simsek O. Synthesis and Characterization of Zn-Doped Mn_3_O_4_ Thin Films Using Successive Ionic Layer Adsorption and Reaction Technique: Its Structural, Optical and Wettability Properties. J. Mater. Sci.: Mater. Electron. 2018, 29 (11), 9466–9473. 10.1007/s10854-018-8980-9.

[ref30] Li D.; Wang Z. R.; Xia Y. M.; Gao Q. L.; Ren M.-M.; Liu W. L.; Kong F. G.; Wang S. J.; Li S. H. Copper-Doped Manganese Tetroxide Composites with Excellent Electrochemical Performance for Aqueous Zinc-Ion Batteries. J. Electroanal. Chem. 2021, 888, 115214 10.1016/j.jelechem.2021.115214.

[ref31] Barai H. R.; Lopa N. S.; Ahmed F.; Khan N. A.; Ansari S. A.; Joo S. W.; Rahman M. M. Synthesis of Cu-Doped Mn_3_O_4_@Mn-Doped CuO Nanostructured Electrode Materials by a Solution Process for High-Performance Electrochemical Pseudocapacitors. ACS Omega 2020, 5 (35), 22356–22366. 10.1021/acsomega.0c02740. 32923793 PMC7482310

[ref32] Chen X.; Chen C.; Xu T.; Xu Y.; Liu W.; Yang W.; Yang P. Performance Enhancement of Asymmetric Supercapacitors with Bud-like Cu-Doped Mn_3_O_4_ Hollow and Porous Structures on Nickel Foam as Positive Electrodes. RSC Adv. 2018, 8 (63), 35878–35887. 10.1039/C8RA06989A. 35558488 PMC9088714

[ref33] Yu M.; Feng X. Thin-Film Electrode-Based Supercapacitors. Joule 2019, 3 (2), 338–360. 10.1016/j.joule.2018.12.012.

[ref34] Scherrer P. Nachr Ges Wiss Goettingen. J. Math. Phys. 1918, 2, 98–100.

[ref35] Prabahar S.; Dhanam M. CdS Thin Films from Two Different Chemical Baths - Structural and Optical Analysis. J. Cryst. Growth 2005, 285 (1–2), 41–48. 10.1016/j.jcrysgro.2005.08.008.

[ref36] Sahay P. P.; Nath R. K. Al-Doped ZnO Thin Films as Methanol Sensors. Sens. Actuators, B 2008, 134 (2), 654–659. 10.1016/j.snb.2008.06.006.

[ref37] Pal M.; Pal U.; Jiménez J. M. G. Y.; Pérez-Rodríguez F. Effects of Crystallization and Dopant Concentration on the Emission Behavior of TiO_2_: Eu Nanophosphors. Nanoscale Res. Lett. 2012, 7, 1–12. 10.1186/1556-276X-7-1. 22214494 PMC3260088

[ref38] Begum A.; Hussain A.; Rahman A. Effect of Deposition Temperature on the Structural and Optical Properties of Chemically Prepared Nanocrystalline Lead Selenide Thin Films. Beilstein J. Nanotechnol. 2012, 3 (1), 438–443. 10.3762/bjnano.3.50. 23016148 PMC3388368

[ref39] Nazir S.; Zhang J. M.; Akhtar M. N.; Abbas N.; Saleem S.; Nauman M.; Ali A. Modification of Physicochemical and Electrical Characteristics of Lead Sulfide (PbS) Nanoparticles (NPs) by Manganese (Mn) Doping for Electronic Device and Applications. J. Sol-Gel Sci. Technol. 2023, 108, 778–790. 10.1007/s10971-023-06176-w.

[ref40] Xu H. Y.; Xu S. L.; Li X. D.; Wang H.; Yan H. Chemical Bath Deposition of Hausmannite Mn_3_O_4_ Thin Films. Appl. Surf. Sci. 2006, 252 (12), 4091–4096. 10.1016/j.apsusc.2005.06.011.

[ref41] Pramitha A.; Hegde S. S.; Bhat B. R.; George S. D.; Sudhakar Y. N.; Raviprakash Y. Properties of Mn_3_O_4_ Thin Film Electrodes Prepared Using Spray Pyrolysis for Supercapacitor Application. Mater. Chem. Phys. 2023, 307, 128213 10.1016/j.matchemphys.2023.128213.

[ref42] Ren L.; Zhou W.; Wang Y.; Meng M.; Wu S.; Li S. Magnetic Properties of Mn_3_O_4_ Film with a Coexistence of Two Preferential Orientations. J. Appl. Phys. 2014, 116 (2), 023906 10.1063/1.4889819.

[ref43] Tomar S. S.; Yadav E.; Soni K.; Mavani K. R. Systematic Effects of Ti Doping on the Electronic Properties of LaNiO_3_ Thin Films. Bull. Mater. Sci. 2021, 44 (2), 118–126. 10.1007/s12034-021-02380-y.

[ref44] Vignesh R.; Sivakumar R.; Sanjeeviraja C. A Detailed Analysis on Optical Parameters of Spinel Structured Mn_3_O_4_ Thin Films Deposited by Nebulized Spray Pyrolysis Technique. Opt. Mater. 2021, 111, 110580 10.1016/j.optmat.2020.110580.

[ref45] Quesne-turin A. J.; Vallverdu G.; Flahaut D.; Croguennec L.; Ménétrier M.; Baraille I.; Quesne-turin A. J.; Vallverdu G.; Flahaut D.; Allouche J. Morphology and Surface Reactivity in the Li_1+x_ Mn_2-x_ O_4_ Spinel with x = 0. 05 and 0. 10: A Combined First Principle and Experimental Study. ACS Appl. Mater. Interfaces 2017, 9, 44922–44930. 10.1021/acsami.7b15249. 29210264

[ref46] Chang F. M.; Brahma S.; Huang J. H.; Wu Z. Z.; Lo K. Y. Strong Correlation between Optical Properties and Mechanism in Deficiency of Normalized Self-Assembly ZnO Nanorods. Sci. Rep. 2019, 9 (1), 905–909. 10.1038/s41598-018-37601-8. 30696935 PMC6351557

[ref47] Zhang S.; Liu G.; Qiao W.; Wang J.; Ling L. Oxygen Vacancies Enhance the Lithium Ion Intercalation Pseudocapacitive Properties of Orthorhombic Niobium Pentoxide. J. Colloid Interface Sci. 2020, 562, 193–203. 10.1016/j.jcis.2019.12.015. 31838355

[ref48] Liu H.; Xie J.; Liu P.; Dai B. Effect of Cu^+^/Cu^2+^ Ratio on the Catalytic Behavior of Anhydrous Nieuwland Catalyst during Dimerization of Acetylene. Catalysts 2016, 6 (8), 120–211. 10.3390/catal6080120.

[ref49] Sivakumar R.; Manisankar P.; Jayachandran M.; Sanjeeviraja C. Intercalation Studies on Electron Beam Evaporated MoO_3_ Films for Electrochemical Devices. Sol. Energy Mater. Sol. Cells 2006, 90 (15), 2438–2448. 10.1016/j.solmat.2006.03.016.

[ref50] Dubal D. P.; Gund G. S.; Holze R.; Jadhav H. S.; Lokhande C. D.; Park C. J. Surfactant-Assisted Morphological Tuning of Hierarchical CuO Thin Films for Electrochemical Supercapacitors. Dalton Trans. 2013, 42 (18), 6459–6467. 10.1039/c3dt50275a. 23471154

[ref51] Suktha P.; Phattharasupakun N.; Dittanet P.; Sawangphruk M. Charge Storage Mechanisms of Electrospun Mn_3_O_4_ Nanofibres for High-Performance Supercapacitors. RSC Adv. 2017, 7 (16), 9958–9963. 10.1039/C6RA28499J.

[ref52] Chen Y. C.; Hsu Y. K.; Lin Y. G.; Lin Y. K.; Horng Y. Y.; Chen L. C.; Chen K. H. Highly Flexible Supercapacitors with Manganese Oxide Nanosheet/Carbon Cloth Electrode. Electrochim. Acta 2011, 56 (20), 7124–7130. 10.1016/j.electacta.2011.05.090.

[ref53] Dong R.; Ye Q.; Kuang L.; Lu X.; Zhang Y.; Zhang X.; Tan G.; Wen Y.; Wang F. Enhanced Supercapacitor Performance of Mn_3_O_4_ Nanocrystals by Doping Transition-Metal Ions. ACS Appl. Mater. Interfaces 2013, 5 (19), 9508–9516. 10.1021/am402257y. 24001053

[ref54] Zhao X.; Du Y.; Li Y.; Zhang Q. Encapsulation of Manganese Oxides Nanocrystals in Electrospun Carbon Nanofibers as Free-Standing Electrode for Supercapacitors. Ceram. Int. 2015, 41 (6), 7402–7410. 10.1016/j.ceramint.2015.02.053.

[ref55] Tholkappiyan R.; Naveen A. N.; Vishista K.; Hamed F. Investigation on the Electrochemical Performance of Hausmannite Mn_3_O_4_ Nanoparticles by Ultrasonic Irradiation Assisted Co-Precipitation Method for Supercapacitor Electrodes. J. Taibah Univ. Sci. 2018, 12 (5), 669–677. 10.1080/16583655.2018.1497440.

[ref56] Chang J. K.; Chen Y. L.; Tsai W. T. Effect of Heat Treatment on Material Characteristics and Pseudo-Capacitive Properties of Manganese Oxide Prepared by Anodic Deposition. J. Power Sources 2004, 135 (1–2), 344–353. 10.1016/j.jpowsour.2004.03.076.

[ref57] Lee E.; Lee T.; Kim B. S. Electrospun Nanofiber of Hybrid Manganese Oxides for Supercapacitor: Relevance to Mixed Inorganic Interfaces. J. Power Sources 2014, 255, 335–340. 10.1016/j.jpowsour.2014.01.011.

[ref58] Beknalkar S. A.; Teli A. M.; Harale N. S.; Patil D. S.; Sutar J. R.; Shin J. C.; Patil P. S. Supercapacitive Performance of SILAR Grown Mn_3_O_4_ Nanoclusters: Effect of Cationic Precursor Concentration. Chin. J. Phys. 2021, 72, 145–158. 10.1016/j.cjph.2021.03.028.

[ref59] Durai G.; Kuppusami P.; Theerthagiri J. Microstructural and Supercapacitive Properties of Reactive Magnetron Co-Sputtered Mo_3_N_2_ Electrodes: Effects of Cu Doping. Mater. Lett. 2018, 220, 201–204. 10.1016/j.matlet.2018.02.120.

[ref60] Sambath Kumar K.; Cherusseri J.; Thomas J. Two-Dimensional Mn_3_O_4_ Nanowalls Grown on Carbon Fibers as Electrodes for Flexible Supercapacitors. ACS Omega 2019, 4 (2), 4472–4480. 10.1021/acsomega.8b03309. 31459642 PMC6648869

[ref61] Vázquez-Olmos A.; Redón R.; Rodríguez-Gattorno G.; Esther Mata-Zamora M.; Morales-Leal F.; Fernández-Osorio A. L.; Saniger J. M. One-Step Synthesis of Mn3O4 Nanoparticles: Structural and Magnetic Study. J. Colloid Interface Sci. 2005, 291 (1), 175–180. 10.1016/j.jcis.2005.05.005. 16005011

