# Supplementary material for: Elucidating the Role of Copper-Induced Mixed Phases on the Electrochemical Performance of Mn-Based Thin-Film Electrodes
Source: ACS Omega. 2023 Nov 30;8(49):46640–52. doi: 10.1021/acsomega.3c05614 (PMC10719916; doi:10.1021/acsomega.3c05614)
Supplement: Supplementary file 1 — ao3c05614_si_001.pdf [file ao3c05614_si_001.pdf]

Supporting Information

Elucidating the Role of Copper-Induced Mixed Phases on the Electrochemical Performance of Mn-based Thin Film Electrodes

Pramitha Adoor.[1], Shreeganesh Subraya Hegde [2], Badekai Ramachandra Bhat [2*], Sudhakar Narahari Yethadka[3], Raviprakash Yeenduguli [1*]

[1,*]*Department of Physics, Manipal Institute ofTechnology, Manipal Academy of Higher Education, Manipal-576104, Karnataka, India*

[2]*Catalysis and Materials Chemistry Laboratory, Department of Chemistry, National Instituteof Technology Karnataka, Surathkal, Mangalore-575025, Karnataka, India*

[3]*Department of Chemistry, Manipal Institute of Technology, Manipal Academy of Higher Education, Manipal-576104, Karnataka, India*

**Corresponding Authors: Dr Raviprakash Y – raviprakash.y@manipal.edu*
Dr Badekai Ramachandra Bhat - ram@nitk.edu.in

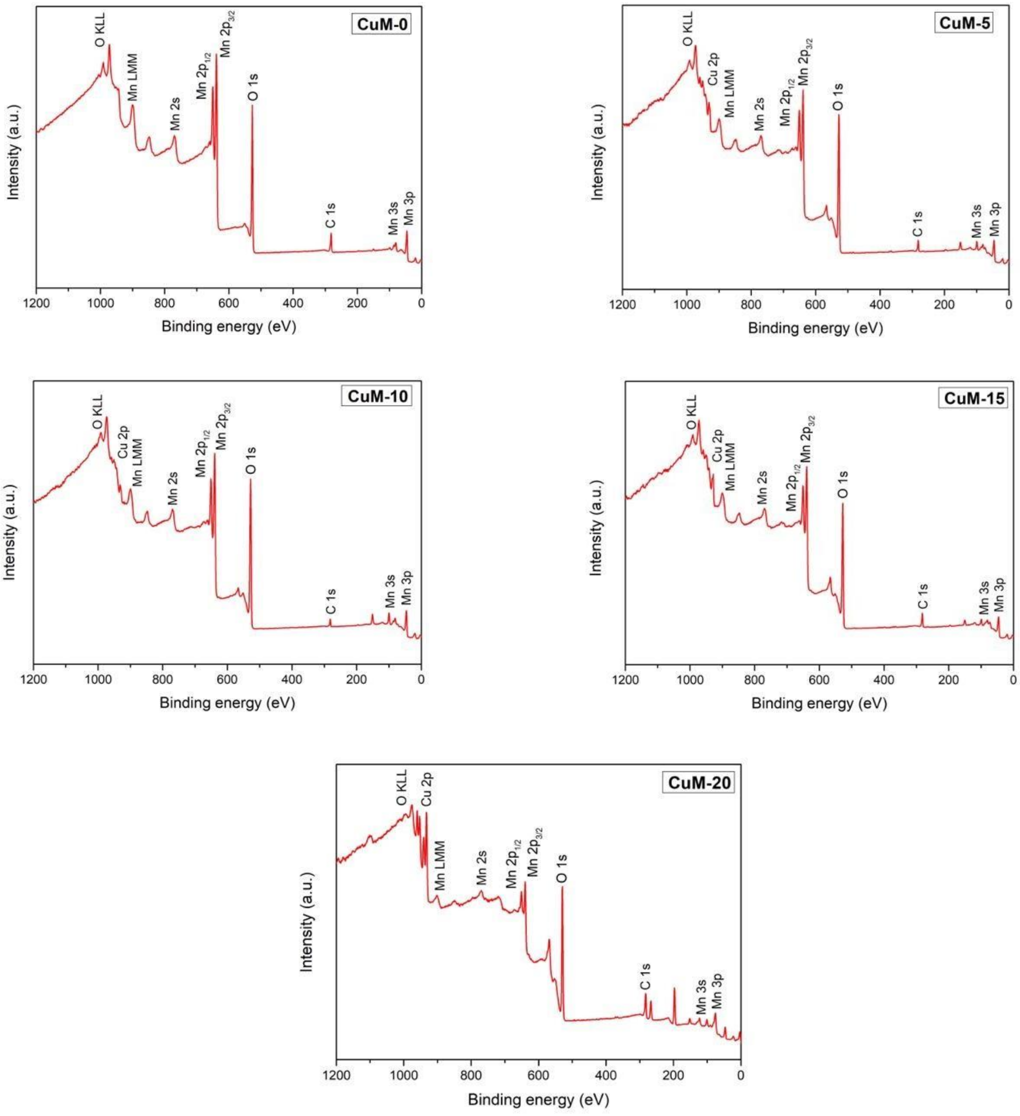

Figure S1: XPS survey spectra of all the samples

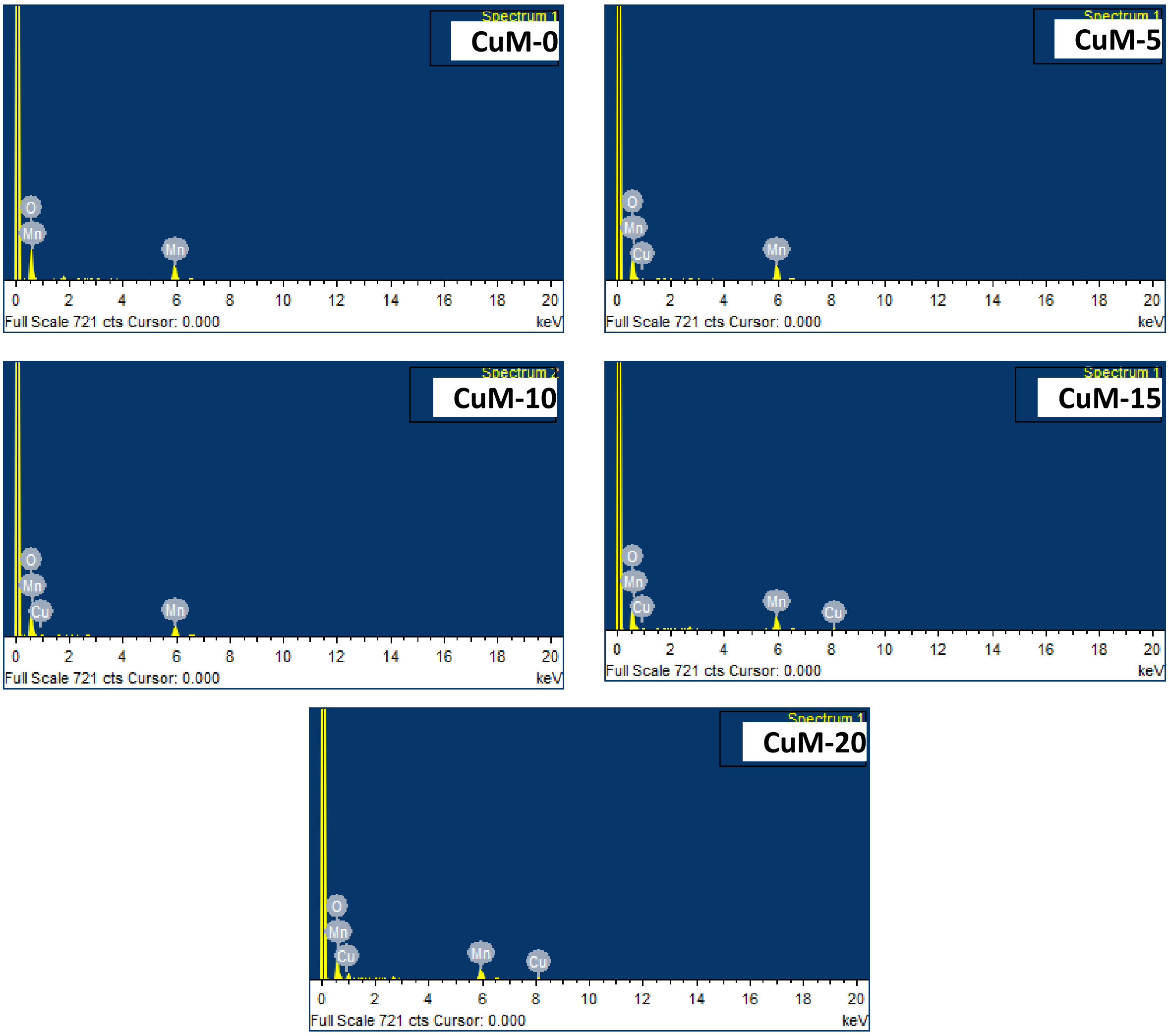

Figure S2: EDS spectra of all the samples
